# An Eventual Sars-CoV-2 Infection Prevention Protocol in the Medical Setting and Dental Office

**DOI:** 10.3390/ijerph18052593

**Published:** 2021-03-05

**Authors:** Luca Fiorillo, Aida Meto, Francesca Cicciù, Rosa De Stefano

**Affiliations:** 1 Department of Biomedical and Dental Sciences, Morphological and Functional Images, School of Dentistry, University of Messina, Policlinico G. Martino, Via Consolare Valeria, 98100 Messina, Italy; 2 Multidisciplinary Department of Medical-Surgical and Odontostomatological Specialties, University of Campania “Luigi Vanvitelli”, 80121 Naples, Italy; 3 Department of Dental Therapy, Faculty of Dental Medicine, University of Medicine, 1005 Tirana, Albania; aidameto@yahoo.com; 4 Unità Operativa di Patologia Clinica, ARNAS Garibaldi Centro, 95123 Catania, Italy; francescacicciu@gmail.com; 5 Department of Biomedical and Dental Sciences, Morphological and Functional Images, University of Messina, 98100 Messina, Italy; rsdestefano@libero.it

The current Coronavirus disease 2019 (COVID-19) pandemic has affected the entire world population, and in particular the medical-health field, especially dentistry. The earliest known cases mainly involved workers from the Wuhan wet market in China. In the first weeks of January 2020, scientists identified pneumonia in these subjects caused by a new coronavirus. The issue of COVID-19 is becoming increasingly controversial in several areas of medicine. Severe acute respiratory syndrome coronavirus 2 (SARS-CoV-2) is a coronavirus strain that causes COVID-19, the latter responsible for the respiratory disease that led to the outbreak of the COVID-19 pandemic; the World Health Organization (WHO) declared this pandemic condition on 11 March 2020 [1,2,3]. SARS-CoV-2 was found to be similar in at least 70% of its gene sequence to that of SARS-CoV [4].

This virus has caused serious confusion around the world in public and social health and the economy. Both symptomatic and asymptomatic patients can transmit the virus by contaminating the air around them through sneezing, coughing and breathing. Unfortunately, the droplets and aerosols created by these infected individuals are inhaled by uninfected individuals. Establishing dental health could be an important pathway for the transmission of infectious diseases from the air or droplets, to both dental staff and patient. As new infectious diseases emerge, infection control protocols in dental care are modified accordingly. In the case of COVID-19, the baseline for taking the necessary dental precautions is that all patients have to be considered positive for SARS-CoV-2, even those who are not yet symptomatic, transmitted when dental health care is provided, primarily through aerosols. In this way, according to many guidelines, the infectious reservoir could be impeded by preventing contact with an infected patient [5,6,7].

During this pandemic, in most countries, dental treatment is limited to provide emergency care and it is presumed that infected patients have to visit the dental office only if strictly needed. As long as health care is essential, especially eliminating infectious reservoirs to prevent transmission, hand and surface hygiene has always been important as a measure against the spread of viruses in any environment, even these are accounted for SARS-CoV-2 [8]. However, to limit the influx of patients to specialized clinics given the need to reduce the risk of COVID-19 infection, in some countries, it has been possible to treat only urgent cases and, in any case, to use specific triage both by telephone and in office to identify the risk related to the patient [5,9,10]. The literature informs us about the possibility of identifying patients affected by COVID-19 also through some oral signs, but in the case of completely asymptomatic patients, it is necessary to rely only on protocols. These, in addition to the use of adequate protection devices, also suggested the creation of particular aspirators or air purifiers [9,10].

A very important role in the prevention of the need for health care is also played by the sterilization and decontamination of medical instruments and the environment. As it is known, cleaning removes the virus and disinfection is able to inactivate it, while surfaces that can be contaminated with SARS-CoV-2 can be effectively disinfected within 1 min by applying at least 62% alcohol, 0.5% hydrogen peroxide (H_2_O_2_) or 0.1% sodium hypochlorite (NaOCl). It is important to note that cleaning and/or disinfection should include all surfaces in the treatment room and any other objects and locations in the dental office that may have been touched by the patient [11]. In addition, effective disinfection has also been reported with various formulations of alcohol-based hand solutions and lower alcohol dilutions [12,13,14].

Several articles have recently been published suggesting that rinsing the mouth with 1% H_2_O_2_ may be helpful in reducing the risk of transmitting SARS-CoV-2 through aerosols. Despite the high viral load in the saliva, tongue, nose or throat, however, the oral cavity will soon be re-infected after rinsing and, thus, povidone-iodine (PVP-I) has been shown to be functional for oral and nasal disinfection anti-SARS-CoV-2. From a systematic review, it was reported that, when rinsing with other types of oral disinfectants, the microbiological load on aerosols created during dental procedures was reduced; however, it is unclear if this reduction is clinically significant in preventing the transmission of SARS-CoV-2. Considering this, in vitro studies demonstrated that chlorhexidine inactive insufficiently SARS-CoV-2 [15,16,17,18].

Furthermore, droplets and aerosols containing the virus eventually land on various surfaces and contaminate them, where the virus can survive for a long time, lasting from hours to days, such as 24 h on cardboard, 2 days on wood and cloth, up to 7 days on outer layer of surgical masks and 7 days on surfaces such as plastic and stainless steel. In this way, these surfaces become a potential tool of virus transmission by contact and, here, it is worth highlighting the importance of hand hygiene [8,19,20,21].

It should be noted that dental personnel working near the oral cavity are more at risk, due to large aerosols created during some dental treatments, especially when using high-velocity drills, ultrasonic scales and/or air-water syringes. Therefore, all personnel should use gloves, mask, protective cloth and cover for eyes and hair, as well as surgical masks, are also recommended. In addition, shoes should be covered, as aerosol slowly settles on surfaces and floors, contaminating them, thus becoming a carrier of the virus [12].

It has been indicated in various studies that aerosol clouds remain around the dental chair for more than 30 min after the completion of the dental procedure. In particular, the dental chair should be covered with thick plastic that can be disinfected before and after the patient’s treatment, while only essential instruments and tools should be exposed in the treatment area. Important is the spraying and wiping technique, which should be used very rigorously, to disinfect all exposed areas, such as the dental chair, surfaces, light and handles. In addition, dental prostheses, impressions or other orthodontic materials must be disinfected as needed. Chlorine-based disinfectants have been shown to be effective as high-level disinfectants and sterilizers; it is indicated that its effective concentration is 2000 mg/L, which can be used to clean high-frequency contact surfaces, but it is important not to exceed this concentration, due to its corrosion. Alcohol (70% ethyl alcohol) can also be used, which is relatively inert and evaporates quickly, leaving the surfaces dried. The antibacterial and antiviral properties of benzalkonium chloride, with or without isopropyl alcohol, have also been demonstrated [22,23,24,25].

Dental units are potential sources of various microorganisms due to the development of microbial biofilms and their permanent release into the treatment water. Depending on the type of dental unit and water system used, the water can be drinking water or from a unit reservoir, which can be filled with distilled water or sterile or non-sterile disinfectant. Therefore, dental units that are connected to the municipal water supply should be immediately converted into reservoir into which water disinfectants can be added. In these cases, chlorinated disinfectants, such as chlorine dioxide and hypochlorous acid, which have been shown to be quite effective, should be considered. The effectiveness of the sterilizer must be monitored and determined using the conventional, biological, chemical or mechanical indicators [26,27,28].

Healthcare facilities have been forced to adopt new and updated protocols to face the risk of Sars-CoV-2 infection; these protocols have also affected dental clinics [8]. The risk seems to be more concentrated, not related to the invasiveness or otherwise of the medical performance, but to the production of aerosols. The possibility, therefore, of nebulizing the patient’s saliva, together with the aerosols produced by dental instruments, in the whole surrounding environment, seems to represent the real risk. We know that the World Health Organization (WHO) has distributed “recipes” to make disinfectant solutions for environments and surfaces based on hydrogen peroxide, alcohol or bleach [12]; however, another formulation has been proposed by Italian Research National Council (CNR) [29], with relatively low costs and this solution is already recommended in dental offices. These solutions are also recommended for use through mechanical nebulizers for sanitary environmental disinfection (Figure 1) [19,30,31,32]. The manuscript entitled “Water Contamination Risk on Dental Office” by M. Cicciù, published in 2020, is highly interesting and in this regard [26]; it concerns all the risks of contamination and infection related to the delivery and discharge pipes of the dental unit and regarding the risk to premises and apartments close to a dental office. The possibility of the latter spreading through could also be considered [33,34,35,36].

Dental units are often equipped with an internal water bowl, to avoid using water from the main water supply. Using internal water in the dental units and inserting solutions of H_2_O_2_ and water into the bowl according to the instructions shown in Table 1 could be a solution to reduce the risk of infection of surfaces and the air and deactivate any presence of the virus already in the oral cavity and in the nebulized solution [30].

H_2_O_2_ in low concentrations does not damage skin or mucous membranes; it is not harmful, even for the surrounding environment, by acting without damaging the materials; on the contrary, it could further sanitize the internal piping of dental units [37]. It is also true that, in this condition, in addition to the protocols for the prevention of contagion, it is also necessary to evaluate the possible damage of the used equipment and the environments. Using such high concentrations of hydrogen peroxide in the internal piping of dental units is not recommended, or in any case, it is not mentioned in any guideline. Some more recent dental chairs appear to be designed for self-disinfection of the internal pipes; these in fact, have a container for H2O2. By inserting 3% hydrogen peroxide, the unit is able to automatically dose this substance in the pipes, according to the required standards [38].

- Some of the standards provide for mixing 20 mL of 3% hydrogen peroxide in one liter of water in the internal circuit bowl of the unit and work with this concentration.
- The dental units also provide for the presence of a small tank for automatic disinfection which involves automatic mixing with the water of the pipes, thus obtaining a concentration of hydrogen peroxide at 3% of 0.06% (automatic disinfection).
- It is also possible to insert 3% hydrogen peroxide in the bowl of the internal piping without dilution, but this provides—after the introduction into the circuits and after the air/water exit from the syringe for about 6–10 s—an action time varying between 10 min and maximum 30 min, before rinsing with distilled water to avoid irreversible damage (manual disinfection) [38].

Unfortunately, the automatic disinfection scheme that is approved and does not cause damage to pipes has not proved to be effective against Sars-Cov-2; nor has the first one. A useful study could concern the testing of different concentrations of hydrogen peroxide, given its limited risks, towards Sars-Cov-2, in order to evaluate the minimum effective concentration and the necessary contact time. Perfecting a protocol could be an excellent aid to reduce the risk of infection in dental offices, in addition to current guidelines [39,40].

While these precautions are good, they are still not enough during this pandemic caused by a highly infectious and easily transmitted virus. An important role in reducing the aerosol load could also be represented by ventilators with HEPA filters, through which it is possible to extract and filter out most of the aerosol. Lastly, as a final message, infection control has always been important in dentistry, especially during epidemics and pandemics of airborne infections where additional precautions are needed; although, over a short period of time, all these measures and modifications have changed our clinical routine and come at an additional cost, they have helped us to protect ourselves and be prepared for similar experiences.

## Figures and Tables

**Figure 1:**
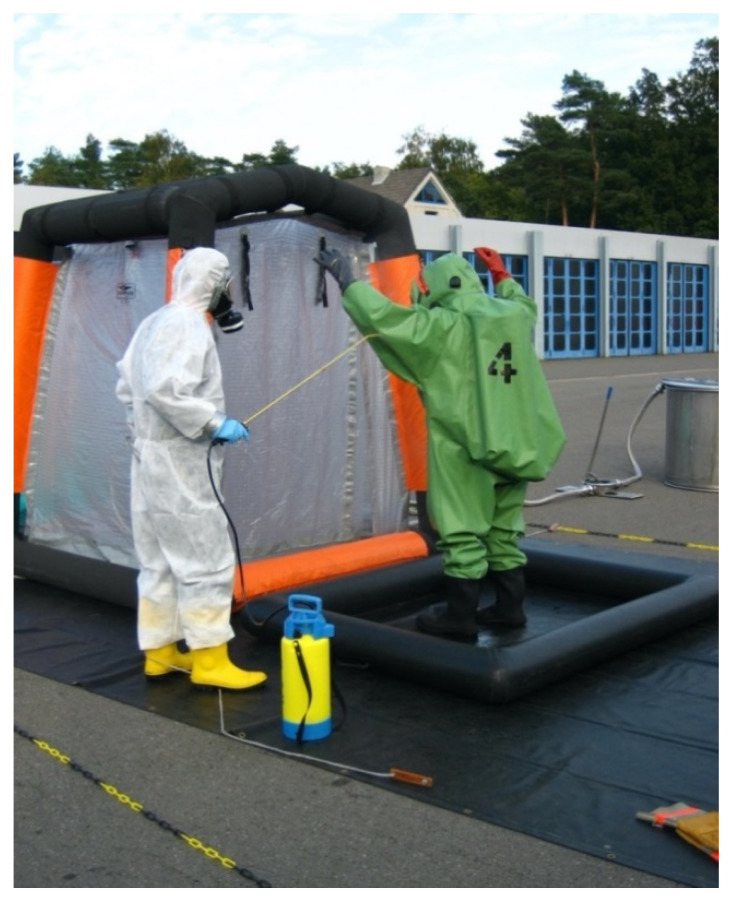
Example of disinfection with mechanical nebulizer (for gentle concession ph. Magnus Mertens) CC BY-SA 2.0 de.

**Table 1:** Disinfectant recipes for environments.

Solution 1	90% Alcohol	0.4 L	0.105669 gal
	Drinkable Water	0.1 L	0.0264172 gal
Solution 2	3% H_2_O_2_	0.1 L	0.0264172 gal
	Drinkable Water	0.4 L	0.105669 gal
Solution 3	5% Bleach	0.01 L	0.002641721 gal
	Drinkable Water	0.499 L	0.13182185 gal

## Data Availability

The data presented in this study are openly available contacting corresponding Author.
